# Complete Genome Sequence of *Chryseobacterium* sp. Strain StRB126, an *N*-Acylhomoserine Lactone-Degrading Bacterium Isolated from Potato Root

**DOI:** 10.1128/genomeA.00952-14

**Published:** 2014-09-25

**Authors:** Tomohiro Morohoshi, Wen-Zhao Wang, Nobutaka Someya, Tsukasa Ikeda

**Affiliations:** a Department of Material and Environmental Chemistry, Graduate School of Engineering, Utsunomiya University, Utsunomiya, Tochigi, Japan; b Institute of Biomedicine and Biotechnology, Shenzhen Institute of Advanced Technology, Chinese Academy of Sciences, Shenzhen, China; c Hokkaido Agricultural Research Center (HARC), National Agriculture and Food Research Organization (NARO), Memuro-cho, Kasai-gun, Hokkaido, Japan

## Abstract

*Chryseobacterium* sp. strain StRB126 was isolated from a potato root and showed *N*-acylhomoserine lactone-degrading activity. Here, we present the complete 5,503,743-bp genome sequence of StRB126, which has a G+C content of 35.6% and carries 4,828 protein-coding genes, six rRNA operons, and 80 tRNA genes.

## GENOME ANNOUNCEMENT

The genus *Chryseobacterium* is a member of the *Cytophaga*-*Flavobacterium*-*Bacteroides* (CFB) group and comprises a heterogeneous group of nonmotile, oxidase-positive, nonfermentative or slowly fermentative Gram-negative bacteria (1). In many Gram-negative bacteria, *N*-acylhomoserine lactones (AHLs) have been identified as signal compounds involved in quorum sensing (2). Many Gram-negative plant pathogens produce AHLs and regulate their virulence by AHL-mediated quorum sensing (3). We have reported that a number of AHL-degrading bacteria were isolated from potato roots, and the dominant isolates were assigned to the genus *Chryseobacterium* (4). We also cloned the *aidC* gene from the genomic library of *Chryseobacterium* sp. strain StRB126, which has AHL-lactonase activity and shows high homology with the metallo-β-lactamase superfamily (5). In a previous study of the genus *Chryseobacterium*, the whole-genome shotgun sequences of *Chryseobacterium gleum* ATCC 35910, have been deposited in DDBJ/EMBL/GenBank databases (accession no. ACKQ02000001 to ACKQ02000007). In this study, we determined the complete genome sequence of *Chryseobacterium* sp. strain StRB126.

Single- and paired-end whole-genome shotgun sequencing of StRB126 was performed using a Roche Genome Sequencer FLX Titanium pyrosequencing technology (6) by Eurofins Genomics (Tokyo, Japan). We produced 896,121 reads with an average read length of 154 bases. The total number of sequenced bases is 138,356,743, representing a sequencing depth of 25×. Using the Celera Assembler version 5.3, these reads were assembled into one large scaffolds including 29 large contigs (>1,000 bp). Gap closure was attempted using gap-spanning clones and PCR products. Prediction of putative coding sequences and gene annotation were done using the Microbial Genome Annotation Pipeline (http://www.migap.org/). Briefly, protein-coding sequences (CDSs) were predicted by the combined use of MetaGeneAnnotator (7), RNAmmer (8), tRNAScan (9), and BLAST (10).

The complete genomic information of the *Chryseobacterium* sp. strain StRB126 is contained on a single circular chromosome of 5,503,743 bp with an average G+C content of 35.6%. The genome contains 4,828 protein-coding genes, six rRNA operons, and 80 tRNA genes. The *aidC* gene (CHSO_3121), which has been identified from the genomic library of StRB126, was found as a single copy in the complete genome. Another predicted coding sequence (CHSO_0423), which encoded 310 amino acids, showed 31.3% identity to the AidC. Many AHL-degrading genes have been cloned and characterized from various bacteria (11). We searched for the homologs of the reported AHL-degrading genes in the complete genome of StRB126. One coding sequence (CHSO_1918), which encoded 522 amino acids, showed partial similarity to the reported AHL-lactonase, AttM/AiiB family from *Agrobacterium tumefaciens* (12). There is the possibility that these genes work as AHL-degrading genes and disrupt quorum sensing in the plant pathogens.

### Nucleotide sequence accession number.

The complete genome sequence of *Chryseobacterium* sp. strain StRB126 has been deposited in the DDBJ/EMBL/GenBank databases under accession no. AP014624.
